# Sinus Floor Elevation Magnitude and Longitudinal Peri-Implant Marginal Bone Change over a Minimum Five-Year Follow-Up: A Retrospective Cohort Study

**DOI:** 10.3390/jcm15208037

**Published:** 2026-10-16

**Authors:** Cristian Niky Cumpătă, Călin Rareș Roman, Mihaela Jana Țuculină, Ioana Smaranda Cumpătă, Alexandru Burcea, Cristina Maria Munteanu, Mădălina Anca Moldovan, Adrian Camen, Sebastian Petrescu, Paolo Di Francesco

**Affiliations:** 1 Department of Oral and Maxillofacial Surgery, Faculty of Dental Medicine, Titu Maiorescu University, 031593 Bucharest, Romania; nikycumpata@yahoo.com; 2 Department of Oro-Maxillo-Facial Surgery and Implantology, Faculty of Dental Medicine, Iuliu Hațieganu University, 400012 Cluj-Napoca, Romania; raresroman25@gmail.com; 3 Department of Endodontics, Faculty of Dental Medicine, University of Medicine and Pharmacy of Craiova, 200349 Craiova, Romania; mtuculina@yahoo.com; 4 Faculty of Dental Medicine, Titu Maiorescu University, 031593 Bucharest, Romania; smarandacumpata@yahoo.com; 5 Department of Speciality Disciplines, Faculty of Dental Medicine, Titu Maiorescu University, 031593 Bucharest, Romania; alexandru.burcea@prof.utm.ro; 6 Department of Oro-Maxillo-Facial Surgery and Implantology, Faculty of Dental Medicine, University of Medicine and Pharmacy, 200349 Craiova, Romania; adrian.camen@umfcv.ro (A.C.); sebastian.petrescu@umfcv.ro (S.P.); 7 Department of Prosthodontics, Faculty of Dental Medicine, Titu Maiorescu University, 031593 Bucharest, Romania; dr.difrancescopaolo@gmail.com

**Keywords:** dental implants, maxillary sinus floor elevation, sinus augmentation, peri-implant marginal bone level, marginal bone change, longitudinal analysis, baseline adjustment, cone-beam computed tomography

## Abstract

**Background/Objectives**: The magnitude of maxillary sinus floor elevation may reflect site anatomy and surgical complexity, but its association with peri-implant marginal bone change remains unclear. This study evaluated whether elevation magnitude was associated with longitudinal peri-implant marginal bone-level change over a minimum 5-year follow-up, distinguishing immediate postoperative differences from subsequent change. **Methods**: This retrospective cohort study included 248 patients with 528 posterior maxillary Dentium SuperLine bone-level implants placed concomitantly with sinus floor elevation. Paired cone-beam computed tomography measurements were available for 522 implants from 247 patients. Elevation magnitude was modeled per 1-mm increment. The primary analysis used a longitudinal mixed-effects model with the time × elevation interaction as the main inferential term. Secondary generalized estimating equation models evaluated baseline and minimum 5-year marginal bone levels after covariate adjustment. **Results**: Greater elevation magnitude was associated with a slightly higher immediate postoperative marginal bone level (B = 0.017 mm per 1-mm increase; 95% CI, 0.005–0.029; *p* = 0.006). The time × elevation interaction was small and not statistically significant (B = 0.004 mm; 95% CI, 0.000–0.008; *p* = 0.066). After adjustment for baseline marginal bone level and the available covariates, no association between elevation magnitude and marginal bone level at the minimum 5-year follow-up was detected (B = 0.000 mm; 95% CI, −0.004 to 0.005; *p* = 0.842). Re-expression on the longitudinal change scale yielded the same estimate, and sensitivity analyses of the secondary models produced similar results. **Conclusions**: Within this retrospective cohort and the clinical protocol used, sinus floor elevation magnitude between 1 and 6 mm was not statistically associated with differential marginal bone-level change over at least 5 years among surviving implants with paired radiographic assessments. This finding should not be interpreted as evidence of equivalence across elevation magnitudes.

## 1. Introduction

The vertical displacement produced during maxillary sinus floor elevation is usually determined by the anatomical conditions of the implant site and by the amount of additional vertical space required for implant placement. Consequently, sinus elevation magnitude is not merely a technical characteristic of the procedure but a quantitative site-specific measure that may reflect differences in preoperative anatomy and surgical complexity. Experimental and clinical studies have shown that the height of membrane displacement or implant protrusion into the sinus does not necessarily translate proportionally into the amount of newly formed bone, suggesting that the biological response within the elevated compartment is influenced by factors other than elevation height alone [1,2].

The relationship between the magnitude of sinus elevation and subsequent bone behavior is biologically complex. The Schneiderian membrane contains cells capable of osteogenic differentiation, while the space created beneath the elevated membrane undergoes progressive organization and mineralization after surgery [3]. At the same time, the geometry of the sinus floor can influence the dimensional stability of the augmented region. Chen et al. demonstrated that sinus floor configuration and implant intrusion angle were associated with subsequent remodeling of the elevated region, indicating that the initial spatial configuration of the surgical site contributes to the radiographic outcome observed during follow-up [4].

An important difficulty in evaluating elevation magnitude is its relationship with the initial anatomy of the treated site. Greater vertical augmentation is frequently required when the available subantral bone is more limited. Thus, the magnitude of elevation may be associated with preoperative anatomical characteristics that can themselves influence the radiographic appearance around the implant. In a 5-year retrospective study of graftless osteotome sinus floor elevation, Zill et al. found that initial residual bone height was associated with apical bone gain, whereas marginal bone loss was not correlated with initial residual bone height [5]. Long-term randomized data have likewise shown that substantial endo-sinus bone formation can remain stable over 5 years in severely atrophic sites, while crestal bone changes remain comparatively limited [6]. These observations indicate that apical bone gain, residual bone anatomy, and marginal peri-implant remodeling represent related but distinct biological phenomena.

Clinical studies also demonstrate that the vertical dimension created by sinus floor elevation is dynamic rather than fixed. Radiographic follow-up has documented reduction in the initially achieved vertical bone height during healing and subsequent remodeling [4,7]. More recent prospective investigations have incorporated sinus floor elevation height together with residual bone height, Schneiderian membrane characteristics, and endo-sinus bone gain, confirming that these parameters describe different components of the augmented site rather than interchangeable measures of the same process [8]. Therefore, a greater elevation height at surgery should not automatically be interpreted as a greater biological challenge to the peri-implant marginal bone compartment or as evidence that greater marginal bone change will subsequently occur.

A second methodological issue concerns the distinction between the marginal bone level observed at a late follow-up examination and the actual change that occurred during follow-up. The final radiographic value contains both the bone level already present at baseline and any subsequent longitudinal modification. This distinction is clinically relevant because early peri-implant bone conditions may be related to later radiographic behavior. In a recent long-term implant study, Moser et al. found that early radiographic bone loss was strongly associated with subsequent progressive bone loss and with less favorable long-term implant outcomes [9]. Accordingly, an association between sinus elevation magnitude and marginal bone level measured several years later cannot, by itself, establish that greater elevation was accompanied by greater longitudinal bone loss. Baseline differences must be considered separately from change over time.

Available clinical studies have predominantly examined residual bone height, endo-sinus bone gain, membrane-related variables, graft remodeling, or implant survival after sinus floor elevation [4,5,6,7,8]. In comparison, the magnitude of sinus elevation itself has received less attention as a quantitative exposure in longitudinal analyses of peri-implant marginal bone level. This distinction may be particularly relevant in cohorts with several years of follow-up, because small differences present immediately after treatment may persist at later examinations and create an apparent gradient in final marginal bone levels even when subsequent rates of change are similar.

Therefore, the primary objective of the present study was to determine whether the magnitude of maxillary sinus floor elevation, modeled quantitatively per 1-mm increment, was associated with the longitudinal evolution of peri-implant marginal bone level from baseline to the minimum 5-year follow-up. Secondary objectives were to evaluate the association between elevation magnitude and marginal bone level at baseline and to determine whether an association with marginal bone level at the minimum 5-year follow-up persisted after accounting for the baseline value and available clinical, surgical, prosthetic, and patient-related covariates. The null hypothesis was that sinus floor elevation magnitude would not be associated with a differential longitudinal change in peri-implant marginal bone level over the minimum 5-year follow-up period.

## 2. Materials and Methods

### 2.1. Study Design and Clinical Setting

A retrospective observational cohort study was conducted using clinical and imaging data from the archive of the Implantology Clinic, Faculty of Dental Medicine, Titu Maiorescu University, Bucharest. Cases of patients treated with implant placement in the posterior maxilla concomitantly with sinus floor elevation between January 2019 and January 2021 were analyzed.

The present study is part of a broader retrospective research project based on the same clinical cohort and comprising three complementary research directions: (1) implant-site preparation and condensation technique, (2) prosthetic retention mode, and (3) sinus floor elevation magnitude. Each direction addresses a distinct primary exposure and research question.

All clinical assessments, treatment planning, and surgical procedures were performed by the same specialist in oral surgery and implantology. Patients were reassessed annually as part of the routine clinical follow-up protocol. According to this protocol, a long-term CBCT examination was scheduled between 60 and 66 months after implant placement. This examination was part of the planned clinical follow-up and was not performed specifically because of symptoms, suspected implant complications, or for the purposes of the present retrospective study. For the present analysis, the CBCT examination performed immediately postoperatively, after implant placement and sinus floor elevation, was considered the baseline radiographic assessment, whereas the routine CBCT examination scheduled at 60–66 months was considered the long-term assessment.

The database was structured at both the patient and implant levels. The same clinical cohort was also used in two other distinct analyses within the broader research project, focusing respectively on implant-site preparation and condensation technique and on prosthetic retention mode. In those analyses, sinus floor elevation magnitude was not the primary exposure; in the prosthetic analysis, it was included only as an adjustment variable. The present study specifically examines sinus floor elevation magnitude as the primary exposure and its relationship with the longitudinal evolution of peri-implant marginal bone level. The main term of interest is the interaction between assessment time and elevation magnitude, used to determine whether bone-level change over time differs with each additional millimeter of elevation. Site-preparation technique, prosthetic characteristics, and the other available clinical factors were included only as adjustment variables or in secondary exploratory analyses. The primary hypothesis, longitudinal modeling strategy, tables, and figures presented in this study were developed specifically for this research question. The study was reported in accordance with the STROBE (Strengthening the Reporting of Observational Studies in Epidemiology) recommendations for observational cohort studies.

### 2.2. Cohort Selection

The retrospective cohort eligible for the present analysis comprised 248 adult patients and 528 Dentium SuperLine implants placed in the posterior maxilla concomitantly with sinus floor elevation. Eligibility criteria were an age of at least 18 years, complete clinical documentation, identification of the technique used for implant-site preparation, availability of information on sinus elevation, a baseline radiographic assessment, and availability of the scheduled long-term follow-up CBCT examination at 60–66 months or documentation of implant loss before that assessment.

Cases with incomplete clinical or imaging documentation, insufficient information regarding the surgical procedure, or no final assessment allowing implant status to be established were excluded. Implants lost before the final radiographic assessment were not included in models requiring a numerical marginal bone-level value at 5 years.

Of the 528 implants in the cohort, 522 implants from 247 patients had comparable radiographic values both at baseline and after at least 60 months and constituted the main sample for the longitudinal analyses. The remaining six implants were documented as lost before the final radiographic assessment. Because the retrospective archive did not allow complete reconstruction of all treated cases that were subsequently excluded before formation of the eligible cohort, the proportion of these losses should not be interpreted as an estimate of implant survival for the entire treated population. There were no additional exclusions due to missing data for covariates included in the main models. Owing to the retrospective nature of the clinical archive, the total number of patients and implants initially assessed before application of the eligibility criteria, as well as the detailed distribution of reasons for exclusion, could not be reliably retrieved. Consequently, the complete cohort-selection flow could not be reconstructed. Although the long-term CBCT examination was scheduled as part of the routine clinical follow-up protocol rather than being obtained for a specific clinical indication, inclusion in the radiographic cohort necessarily required availability of this examination. Patients without an available long-term follow-up CBCT could therefore not contribute to the longitudinal radiographic analysis. Because the retrospective archive did not allow reliable reconstruction of how many initially treated patients lacked this examination or the reasons for its absence, availability-related selection bias cannot be excluded.

Because the study retrospectively used an available clinical cohort, no a priori sample-size calculation was performed. The analytical sample size was determined by the eligible cases available in the archive for which the data required for the objectives of the present analysis were available. Accordingly, interpretation of the results was based on the magnitude of the estimates and the 95% confidence intervals, and lack of statistical significance was not considered evidence of equivalence or absence of an effect.

### 2.3. Surgical and Implant Protocol

Preoperative planning was performed using cone-beam computed tomography, with assessment of residual bone dimensions, the relationship to the maxillary sinus floor, and the three-dimensional implant position established according to prosthetic requirements. Procedures were performed under local anesthesia after elevation of a full-thickness mucoperiosteal flap.

Dentium SuperLine implants with diameters of 4.0 or 4.5 mm and lengths of 8, 10, or 12 mm were used. Implants were placed simultaneously with sinus floor elevation in premolar or molar sites of the posterior maxilla. Implant dimensions were selected according to the available bone volume, sinus anatomy, planned prosthetic position, and the possibility of achieving primary stability.

Selection of the sinus approach took into account a residual bone height of at least 4 mm, a crestal width of at least 5 mm, and the planned elevation magnitude. The transcrestal approach was used for limited elevation of approximately 3 mm. For elevations greater than 3 mm, the lateral approach was used. The same patient could have sites treated with different approaches depending on local anatomy.

Implant-site preparation and condensation were performed using osteotomes, Densah/Versah burs, or the DASK Simple system, according to the clinical indication and local anatomy. In the present study, these techniques were not the exposure of interest and were entered into the statistical models only as adjustment variables.

Augmentation material was used for both the transcrestal and lateral approaches. In transcrestal procedures, THE Graft xenograft (Purgo Biologics, Seongnam, Gyeonggi-do, Republic of Korea); particle size 1.0–2.0 mm) was progressively inserted after membrane elevation. In lateral procedures, the same material was used in combination with platelet-rich fibrin. The amount of material was adapted to the anatomy and elevation magnitude and could not be quantified retrospectively in a uniform manner for all implants.

All implants were either immediately restored or immediately loaded.

Primary implant stability was assessed by insertion torque and, when available, by resonance frequency analysis. Immediate restoration without static or dynamic occlusal contacts was used in situations other than All-on-X rehabilitations, whereas immediate loading with controlled functional contacts was used exclusively for splinted All-on-X rehabilitations. Provisional restorations were delivered within the first 24–72 h, and definitive restorations after 6–8 months. Prosthetic treatment characteristics were considered adjustment covariates and were not exposures of interest in the present analysis.

### 2.4. Sinus Floor Elevation Magnitude

The primary exposure variable was the vertical magnitude of sinus floor elevation, expressed in millimeters and determined at the individual implant level. For each implant site, the preoperative and immediate postoperative CBCT datasets were reviewed together. The preoperative examination was used to identify the native position and local contour of the maxillary sinus floor at the planned implant site. The corresponding postoperative volume was evaluated using multiplanar reconstruction and reoriented according to the longitudinal axis of the placed implant. Correspondence between the preoperative and postoperative sections was established by visual comparison of the planned implant site and the local configuration of the native sinus floor with the position of the implant on the postoperative examination. No software-based image registration or automated superimposition of the two CBCT datasets was used.

The native sinus floor identified on the preoperative CBCT served as the anatomical reference for its original position. On the immediate postoperative reconstruction, elevation magnitude was measured parallel to the longitudinal axis of the implant, from this reference level to the most apical limit of the elevated sinus floor. Thus, the preoperative dataset was used to establish the original sinus-floor reference, whereas the numerical measurement was obtained on the immediate postoperative CBCT. Values were recorded at the implant level directly as integer values in 1-mm increments, ranging from 1 to 6 mm. No separate unrounded continuous measurements were retained in the clinical database. The retrospective records did not allow reliable determination of whether these integer values had originally been obtained by rounding to the nearest millimeter, truncation, or assignment to predefined 1-mm intervals. No additional rounding, truncation, or categorization was performed for the present statistical analysis.

For clinical description of the cohort and secondary exploratory analyses, elevation magnitude was additionally classified into three categories: low, 1–2 mm; moderate, 3–4 mm; and high, 5–6 mm. However, the primary predictor was retained in numerical form, expressed in millimeters, to avoid the loss of information associated with grouping values into categories.

The shape of the relationship between elevation magnitude and bone outcomes was assessed by comparing the linear model with a model that additionally included a quadratic elevation term. Elevation-magnitude measurements were independently reviewed by all three clinicians experienced in implantology and CBCT interpretation. Both the preoperative and immediate postoperative datasets were available during this assessment because identification of the original sinus-floor reference required visual comparison between the two examinations. When discrepancies occurred in section correspondence, identification of the native sinus-floor reference, or the resulting measurement, the datasets were jointly reassessed and the final value was established by consensus. A separate inter-examiner reproducibility assessment was performed on a random subset of 60 implant sites. Sinus floor elevation magnitude was independently measured by each of the three clinicians using the same measurement protocol. Because nonlinear modeling did not improve model fit, the predictor was modeled linearly on the numerical scale in the main analyses.

### 2.5. Radiographic Assessment of Marginal Bone Level

The baseline radiographic assessment of marginal bone level was the CBCT examination performed immediately postoperatively, after implant placement and completion of the sinus elevation procedure. The final radiographic assessment corresponded to the routine long-term CBCT examination scheduled between 60 and 66 months after implant placement as part of the clinical follow-up protocol. These examinations were not acquired specifically for the present retrospective analysis or in response to a particular clinical indication. No additional CBCT examinations were performed for study purposes. Hereafter, for concision, this examination is referred to as the “5-year assessment”.

Baseline and long-term follow-up CBCT examinations were performed at the same clinic using the same CBCT system, Veraview X800 (J. MORITA MFG. CORP., Kyoto, Japan), and the same acquisition protocol. The acquisition settings were a field of view of 80 × 80 mm, voxel size of 0.125 mm, tube voltage of 80 kV, tube current of 6 mA, exposure time of 9.4 s, and 180° rotation.

CBCT data were exported in DICOM format and analyzed using Romexis software (version 7, Planmeca Oy, Helsinki, Finland). For each examination, the volume was reoriented by aligning the longitudinal axis of the implant in the multiplanar reconstruction planes to obtain comparable sections at the two assessment time points.

The same Romexis software platform and measurement workflow were used for evaluation of the baseline and follow-up datasets. However, because of the retrospective design and the duration of the clinical follow-up, the exact software version used at each examination and the original reconstruction-related settings, including fixed window-level and metal-artifact-reduction settings, were not systematically archived and could not be reliably reconstructed. Accordingly, identical acquisition parameters can be confirmed, whereas identity of all reconstruction and software-version parameters across examinations cannot be established retrospectively.

Peri-implant marginal bone level was determined separately on the mesial and distal surfaces. For each surface, the linear distance between the implant shoulder and the most coronal radiographically visible bone–implant contact was measured. The value used in implant-level analyses was the arithmetic mean of the mesial and distal measurements.

Marginal bone-level measurements were independently assessed by the same three clinicians. During these assessments, the examiners were aware of the examination time point, baseline or 5-year follow-up, but did not have access to the recorded sinus floor elevation magnitude. When discrepancies occurred in landmark identification or measurement, the images were jointly reassessed and a final consensus value was established. The values entered into the analytical dataset were the final consensus measurements. A separate inter-examiner reproducibility assessment was performed on the same random subset of 60 implant sites. Each of the three clinicians independently measured marginal bone level at the immediate postoperative baseline and at the 5-year assessment using the same measurement protocol. Formal intra-examiner reproducibility could not be assessed because repeated measurements by the same examiner in a separate assessment session were not available.

### 2.6. Definition of Outcomes and Covariates

The outcome used in the primary longitudinal analysis was peri-implant marginal bone level, expressed in millimeters and assessed at two time points: baseline and after a minimum of 5 years.

Longitudinal change in marginal bone level was calculated for each implant as:

Δ *marginal bone level* = *marginal bone level at* 5 *years* − *baseline marginal bone level*.

A positive value indicated apical displacement of the most coronal bone–implant contact between the two examinations, whereas a value close to zero indicated longitudinal radiographic stability. In the present study, the term “marginal bone-level change” was preferred to the broader term “bone loss” because the calculated value describes the radiographic difference between two time points and may include changes of very small magnitude. Implant loss before the final radiographic assessment was reported separately as a secondary clinical outcome and was not combined with radiographic marginal bone-level values.

At the patient level, age, female/male category, smoking status, history of periodontitis, and number of implants per patient were available. History of periodontitis was defined as a documented diagnosis of periodontitis that had been established and treated before implant placement. Thus, this covariate did not denote untreated active periodontal disease at the time of implant surgery. The classification was based on the periodontal diagnosis recorded in the clinical documentation before implant treatment. The specific diagnostic criteria used at the time of the original periodontal diagnosis could not be reconstructed consistently from the retrospective records. Detailed information on periodontal stage, grade, probing-depth distribution, and clinical attachment level was not available in a sufficiently standardized form for all patients and therefore could not be incorporated into the retrospective analysis. At the implant level, sinus floor elevation magnitude, implant-site preparation and condensation technique, baseline and 5-year marginal bone levels, provisional and definitive restoration retention, and the immediate protocol, classified as immediate restoration or immediate loading, were recorded.

### 2.7. Statistical Analysis

Statistical analyses were performed using IBM SPSS Statistics for Windows, version 32.0 (IBM Corp., Armonk, NY, USA). Quantitative variables were described using mean, standard deviation, median, interquartile range, and minimum–maximum range, whereas categorical variables were described using absolute frequencies and percentages. Patient-level characteristics were summarized using one record per unique patient. For descriptive summaries stratified by elevation magnitude, each patient was counted once within each elevation category in which at least one implant was represented. Thus, patients contributing implants to more than one elevation category could appear in more than one category, but no patient was counted more than once within the same category. Implant-level clinical, procedural, and radiographic characteristics were summarized separately using the implant as the unit of description. To characterize covariate imbalance across the three elevation-magnitude categories, absolute standardized differences were calculated for the pairwise comparisons of low versus moderate, low versus high, and moderate versus high elevation. For continuous variables, standardized differences were based on the difference in means divided by the pooled standard deviation; for binary variables, they were based on the difference in proportions standardized by the pooled binomial variance. Standardized differences for patient-level characteristics were calculated using the unique-patient summaries described above, whereas standardized differences for implant-level characteristics used the implant as the unit of description. These measures were used descriptively and were not interpreted as hypothesis tests. Inter-examiner reproducibility was evaluated for sinus floor elevation magnitude, immediate postoperative marginal bone level, and 5-year marginal bone level in the random subset of 60 implant sites independently assessed by the three clinicians. Reliability was quantified using a two-way random-effects, absolute-agreement, single-measure intraclass correlation coefficient [ICC(2,1)]. Ninety-five percent confidence intervals were obtained by bootstrap resampling of implant sites. Measurement error was quantified using the standard error of measurement (SEM), calculated as SD × √(1 − ICC), and the 95% minimal detectable change (MDC95), calculated as 1.96 × √2 × SEM. In the main inferential analyses, sinus floor elevation magnitude was used as a quantitative predictor modeled linearly, with the effect expressed for each additional 1 mm. Categories of 1–2 mm, 3–4 mm, and 5–6 mm were used in secondary exploratory analyses, whereas the main models retained elevation magnitude on the numerical scale without categorization.

Actual follow-up duration was calculated for each implant as the interval, in months, between the immediate postoperative baseline CBCT and the final long-term CBCT examination. Follow-up duration was summarized using the mean, standard deviation, median, interquartile range, and minimum–maximum range. The distribution of follow-up duration was also described across the low-, moderate-, and high-elevation categories and compared using the Kruskal–Wallis test. Its association with elevation magnitude on the numerical 1–6 mm scale was additionally examined using Spearman’s rank correlation coefficient.

Given the asymmetric distribution of marginal bone level and the high proportion of values equal to 0 mm, unadjusted exploratory relationships between sinus floor elevation magnitude and baseline marginal bone level, marginal bone level at 5 years, and longitudinal change were assessed using Spearman’s rank correlation coefficient. Differences among the three clinical elevation categories were evaluated using the Kruskal–Wallis test. Pairwise comparisons were performed using the Mann–Whitney U test with Holm adjustment for multiple comparisons. The proportions of implants with a detectable marginal bone level (>0 mm) at 5 years were compared exploratorily among the three elevation categories using Pearson’s χ^2^ test. The proportion of implants with a positive longitudinal change (>0 mm) was reported descriptively for the three elevation-magnitude categories. The distribution of implants lost before the final assessment was compared exploratorily among the three magnitude categories using the Fisher–Freeman–Halton exact test. Given the small number of events, implant loss was analyzed descriptively and was not used to construct a multivariable model.

The distribution of marginal bone level at 5 years was also evaluated exploratorily across the six exact elevation values, from 1 to 6 mm, using the Kruskal–Wallis test.

For exploratory assessment of the ordinal trend between the three magnitude categories and bone outcomes, the categories were coded in ascending order and analyzed using Spearman’s rank correlation coefficient.

These unadjusted analyses were exclusively exploratory and did not model the dependence among implants belonging to the same patient; accordingly, the associated *p* values were used to describe the data structure and not for the primary inference.

The primary analysis used a linear mixed model for repeated measures. The sample included 522 implants from 247 patients, with each implant contributing two observations, yielding 1044 longitudinal observations. The dependent variable was peri-implant marginal bone level. Fixed effects included assessment time, sinus floor elevation magnitude, and the time × elevation magnitude interaction. The time × elevation magnitude interaction was the main inferential term of the study because it directly tests whether longitudinal change in marginal bone level from baseline to the minimum 5-year assessment differs with each additional millimeter of elevation.

The longitudinal model was adjusted for implant-site preparation technique, definitive restoration retention, provisional restoration retention, immediate loading versus immediate restoration protocol, age, female/male category, smoking, history of periodontitis, and number of implants per patient. The random-effects structure included an intercept for patient and an intercept for implant nested within patient to account for both dependence among implants belonging to the same patient and the repeated nature of the two measurements for the same implant. The surgical approach, transcrestal or lateral, was not entered as a separate covariate in the main models because its selection was clinically linked to the required elevation magnitude according to the surgical protocol. Under these conditions, the independent effects of approach and elevation magnitude cannot be clearly separated within this cohort. This procedural dependence was considered in the interpretation of the results and treated as a potential source of residual confounding. As a sensitivity analysis, the primary longitudinal mixed-effects model was repeated using the actual elapsed follow-up time for each implant instead of a common binary final-assessment indicator. Time was set to 0 at the immediate postoperative baseline and expressed in years at the final examination according to the individual follow-up interval. The same adjustment covariates and random-effects structure were retained. The actual follow-up time × elevation magnitude interaction was used to assess whether variation in follow-up duration materially affected the primary longitudinal finding. Residual bone height, sinus geometry, Schneiderian membrane thickness, and the exact amount of augmentation material were not uniformly available and could not be included in the adjusted models. Because these factors are closely related to the anatomical indication for sinus elevation and to the required elevation magnitude, substantial residual confounding by baseline anatomy and procedural characteristics remains possible. Accordingly, coefficients for elevation magnitude were interpreted as conditional associations within this observational cohort rather than as estimates of the isolated biological effect of increasing sinus elevation by 1 mm.

To separate differences already present at baseline from the value observed after 5 years, secondary generalized estimating equation (GEE) models were constructed using a Gaussian distribution, an exchangeable correlation structure, and robust standard errors, with patient used as the clustering unit. The first model evaluated marginal bone level at 5 years and included baseline bone level as a mandatory adjustment covariate, together with the available clinical, surgical, and prosthetic covariates.

A separate GEE model evaluated the association between sinus floor elevation magnitude and baseline marginal bone level. This model was adjusted for implant-site preparation technique, immediate protocol, age, female/male category, smoking, history of periodontitis, and number of implants per patient.

Directly calculated longitudinal change, Δ marginal bone level, was additionally analyzed using a Gaussian GEE model with the same clustering structure and the same set of covariates as the 5-year marginal bone-level model. Because Δ was defined as the difference between the 5-year and baseline values and baseline level was included as an adjustment variable, this analysis represents a reparameterization of the baseline-adjusted model and was used to express the result on the longitudinal change scale, rather than as an independent confirmatory analysis.

The shape of the relationship between elevation magnitude and bone outcomes was assessed by introducing a quadratic elevation term and comparing the linear and nonlinear specifications using AIC and BIC, together with nested-model testing. Collinearity among predictors was assessed using the variance inflation factor and tolerance.

In an additional exploratory effect-modification analysis, the interaction between implant-site preparation technique and sinus floor elevation magnitude was tested separately for marginal bone level at 5 years and longitudinal change.

Given the asymmetric distribution of bone outcomes and the high proportion of values equal to 0 mm, the robustness of elevation-magnitude estimates from the secondary models for marginal bone level at 5 years and longitudinal change was additionally assessed using linear regression with patient-clustered robust standard errors and patient-clustered bootstrap with 2000 resamples. Logistic regression for 0 mm versus >0 mm was not used in the primary elevation-magnitude analysis because adjustment for baseline level could generate complete or near-complete separation and unstable estimates.

Model results were expressed as B coefficients, standard errors, 95% confidence intervals, and *p* values. Statistical significance was set at *p* < 0.05, with coefficients interpreted together with effect magnitude and confidence intervals. The time × elevation magnitude interaction in the linear mixed model constituted the primary inferential analysis. The GEE models for baseline marginal bone level, the minimum 5-year level adjusted for baseline, and the reparameterization on the longitudinal change scale were considered secondary analyses. Unadjusted correlations, comparisons among elevation categories and among the exact 1–6 mm values, analysis of the proportion of implants with a detectable marginal bone level, analysis of implant losses, evaluation of the quadratic term, and effect-modification analysis by preparation technique were exploratory. Regressions with robust standard errors and the clustered bootstrap were used exclusively as sensitivity analyses. Holm adjustment was applied to pairwise comparisons; no global multiplicity adjustment was applied to the other secondary and exploratory analyses, and the corresponding *p* values were interpreted in an exploratory context rather than as independent confirmatory evidence.

### 2.8. Ethical Considerations

The study was conducted in accordance with the principles of the Declaration of Helsinki and was approved by the Ethics Committee of Titu Maiorescu University, Bucharest, under Decision No. 3 of 20 January 2017. Informed consent was obtained from all participants in accordance with institutional requirements. The present retrospective analysis did not involve any additional radiographic exposure beyond the examinations already available in the clinical archive.

## 3. Results

### 3.1. Cohort Characteristics and Distribution of Sinus Floor Elevation Magnitude

The overall cohort included 248 patients and 528 implants. Sinus floor elevation magnitude ranged from 1 to 6 mm. Of the 528 implants, 205 (38.8%) were included in the low-elevation category (1–2 mm), 261 (49.4%) in the moderate-elevation category (3–4 mm), and 62 (11.7%) in the high-elevation category (5–6 mm). The distribution of exact values was 54 implants at 1 mm, 151 at 2 mm, 127 at 3 mm, 134 at 4 mm, 45 at 5 mm, and 17 at 6 mm.

Of the 528 implants, 522 from 247 patients had radiographic data available for the bone analyses. The six implants lost before the final assessment did not contribute to models requiring a radiographic value at 5 years.

Among the 522 implants included in the radiographic analyses, the actual follow-up duration was 63.94 ± 1.06 months, with a median of 64 months (IQR, 63–65) and a range of 63–66 months. Follow-up duration was 63.32 ± 0.66 months in the low-elevation group, 64.24 ± 1.12 months in the moderate-elevation group, and 64.78 ± 0.67 months in the high-elevation group. The distribution differed significantly across categories (Kruskal–Wallis H = 144.025; *p* < 0.001), and elevation magnitude was positively correlated with follow-up duration (Spearman ρ = 0.658; *p* < 0.001).

**Figure 1:**
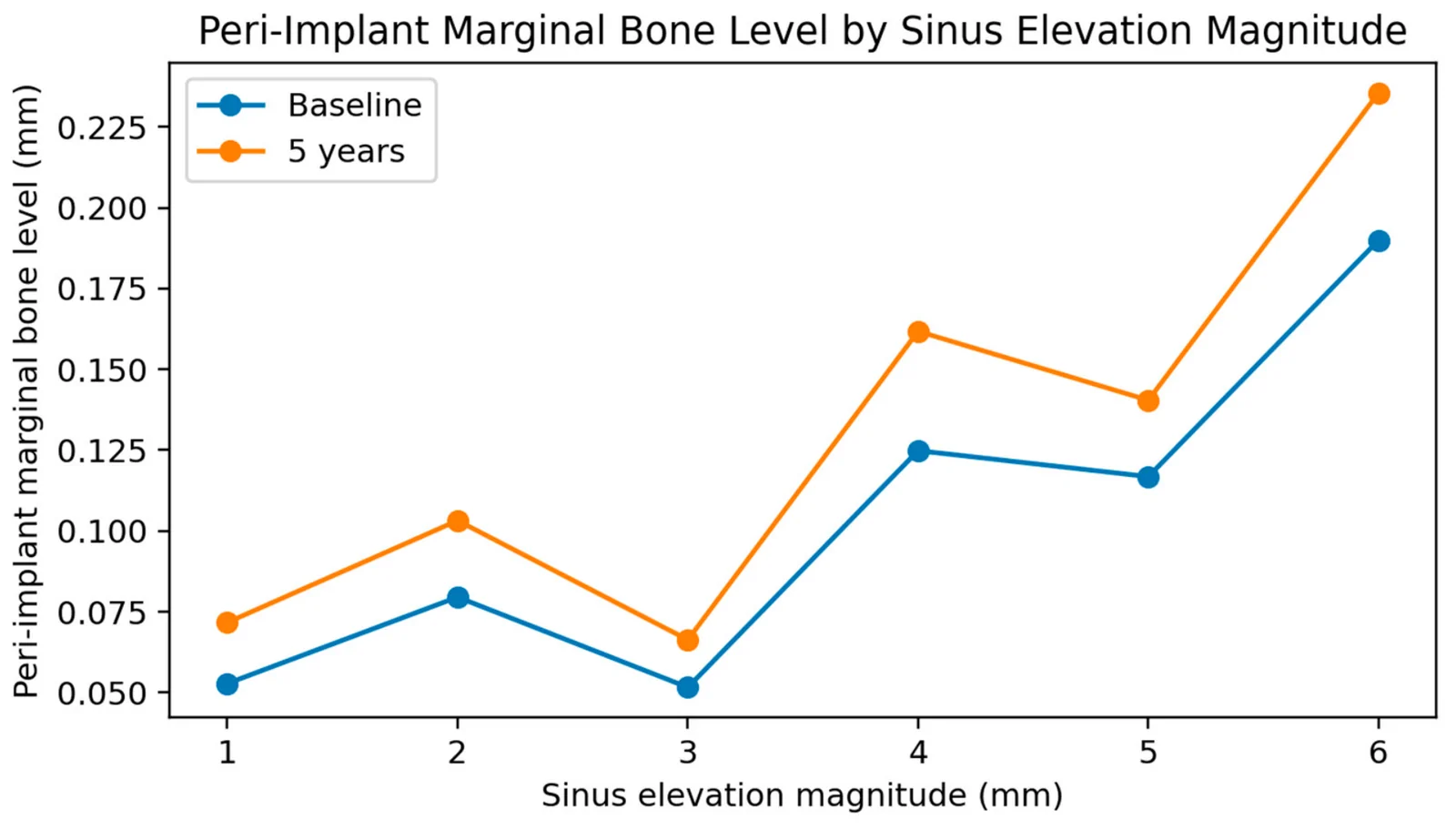
Peri-implant marginal bone level at baseline and 5 years according to sinus floor elevation magnitude.

The six losses occurred in six different patients and were distributed as two in each elevation-magnitude category. The proportions of implants documented as lost within the eligible cohort were 1.0% (2/205) for elevations of 1–2 mm, 0.8% (2/261) for elevations of 3–4 mm, and 3.2% (2/62) for elevations of 5–6 mm. The distribution of these losses across categories did not differ significantly according to the Fisher–Freeman–Halton exact test (*p* = 0.259). Patient-level characteristics, with each patient represented only once within each applicable elevation-magnitude category, are summarized in Table 1. Implant-level clinical, procedural, and radiographic characteristics according to elevation magnitude are presented separately in Table 2. Baseline and 5-year marginal bone levels according to elevation magnitude are illustrated in Figure 1.

Inter-examiner reproducibility was assessed in a random subset of 60 implant sites independently evaluated by the three clinicians. For sinus floor elevation magnitude, the absolute-agreement single-measure ICC was 0.687 (95% bootstrap CI, 0.605–0.743), with an SEM of 0.512 mm and an MDC95 of 1.418 mm. For immediate postoperative marginal bone level, the ICC was 0.649 (95% bootstrap CI, 0.556–0.712), with an SEM of 0.055 mm and an MDC95 of 0.153 mm. For marginal bone level at the 5-year assessment, the ICC was 0.628 (95% bootstrap CI, 0.498–0.717), with an SEM of 0.047 mm and an MDC95 of 0.131 mm.

Pairwise absolute standardized differences for the main baseline characteristics are presented in Table 1 and Table 2. The largest imbalances were observed for the immediate protocol (maximum absolute standardized difference = 0.711), number of implants per patient (0.535), and history of periodontitis (0.524). Smoking showed a smaller imbalance (0.256), whereas implant-site preparation technique showed little between-category imbalance, with maximum absolute standardized differences ≤ 0.091.

### 3.2. Marginal Bone Level According to Exact Sinus Floor Elevation Magnitude

In the descriptive analysis according to exact magnitude values, mean marginal bone levels at 5 years were 0.07 mm for 1-mm elevations, 0.10 mm for 2 mm, 0.07 mm for 3 mm, 0.16 mm for 4 mm, 0.14 mm for 5 mm, and 0.24 mm for 6 mm. In contrast, mean longitudinal change remained small at all magnitude values, ranging from 0.01 to 0.05 mm. Detailed descriptive values according to exact elevation magnitude are presented in Table 3.

In the unadjusted exploratory comparison across the six exact sinus floor elevation-magnitude values, the distribution of marginal bone level at 5 years differed significantly (Kruskal–Wallis, *p* < 0.001). However, the relationship was not strictly monotonic, with mean values of 0.07, 0.10, 0.07, 0.16, 0.14, and 0.24 mm for elevations of 1, 2, 3, 4, 5, and 6 mm, respectively. The smaller number of implants at 5 and 6 mm warrants caution when interpreting differences at the extremes of the distribution.

### 3.3. Unadjusted Exploratory Associations Between Elevation Magnitude and Bone Parameters

The exploratory analysis per exact millimeter showed positive but very small associations between sinus floor elevation magnitude and all three radiographic parameters analyzed. Spearman’s ρ was 0.155 for baseline marginal bone level (*p* < 0.001), 0.148 for marginal bone level at 5 years (*p* < 0.001), and 0.099 for longitudinal change (*p* = 0.023). Thus, the association was already present at baseline and remained of similar magnitude for the final value, whereas the relationship with the change occurring during follow-up was weaker.

When elevation magnitude was analyzed using the three clinical categories, the overall differences did not reach statistical significance for baseline marginal bone level (Kruskal–Wallis H = 5.466; *p* = 0.065), marginal bone level at 5 years (H = 3.848; *p* = 0.146), or longitudinal change (H = 1.262; *p* = 0.532). Ordinal trend analysis showed a weak association for the baseline value (ρ = 0.094; *p* = 0.031), but not for the 5-year value (ρ = 0.082; *p* = 0.060) or longitudinal change (ρ = 0.049; *p* = 0.262). Pairwise comparisons among the low-, moderate-, and high-elevation categories did not remain significant after Holm adjustment.

The proportion of implants with a detectable marginal bone level (>0 mm) at 5 years was 32.5% in the low-elevation category, 35.9% in the moderate category, and 40.0% in the high category. The difference among the three categories was not statistically significant (χ^2^, *p* = 0.521). Similarly, the proportion of implants with a positive longitudinal change was 18.2%, 20.8%, and 23.3%, respectively, across the three elevation categories. These percentages are presented descriptively.

### 3.4. Primary Longitudinal Model

The linear mixed model included 522 implants, 247 patients, and 1044 longitudinal observations. The model converged appropriately.

The main coefficient for assessment time was B = 0.013 mm (95% CI, −0.001 to 0.027; *p* = 0.076). In the parameterization used, this coefficient corresponds to the estimated temporal change at an elevation magnitude of 0 mm, a value outside the observed 1–6 mm range in the cohort, and therefore has no direct clinical interpretation. Longitudinal interpretation was based primarily on the time × elevation magnitude interaction and on estimated marginal values for clinically observed magnitudes. In the adjusted model, a 1-mm greater sinus floor elevation magnitude was associated with a 0.017-mm higher baseline marginal bone-level value (95% CI, 0.005–0.029; *p* = 0.006).

The primary result of the study was the time × sinus floor elevation magnitude interaction. Its coefficient was B = 0.004 mm for each additional 1 mm of elevation (95% CI, 0.000–0.008; *p* = 0.066). These coefficients represent associations conditional on the measured covariates and should not be interpreted as isolated biological effects of increasing sinus elevation by 1 mm. The primary longitudinal model is summarized in Table 4.

Confidence-interval limits are presented rounded to three decimal places; for the time × elevation magnitude interaction, the lower limit of the 95% CI rounds to 0.000, whereas the coefficient test yielded *p* = 0.066.

The model was adjusted for implant-site preparation technique, definitive and provisional restoration retention, immediate protocol, age, female/male category, smoking, history of periodontitis, and number of implants per patient.

Model indices were AIC = −1844.11; BIC = −1759.94; patient-level ICC = 0.298; total ICC for the random effects = 0.924; marginal R^2^ = 0.048; and conditional R^2^ = 0.927.

**Figure 2:**
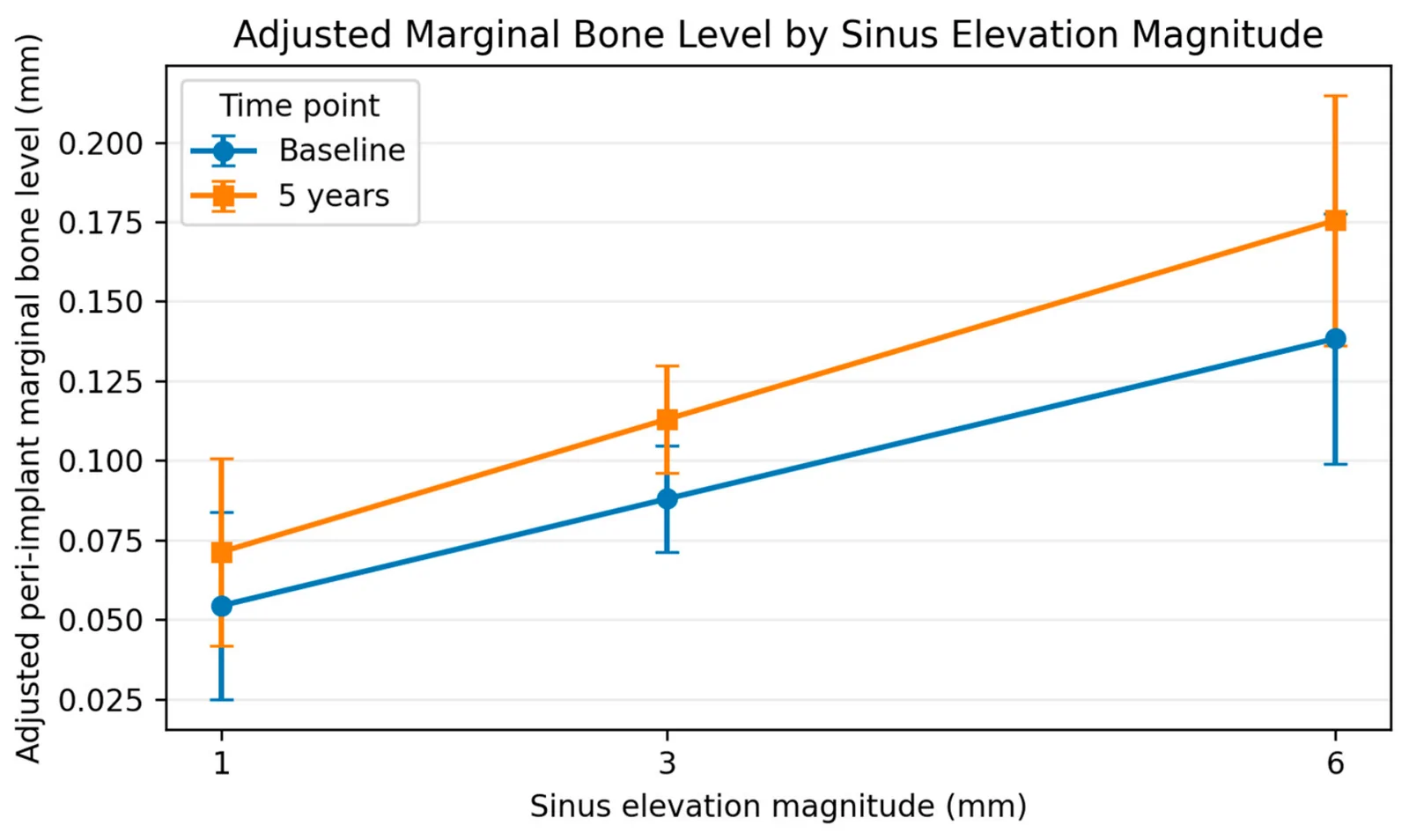
Adjusted marginal bone level at baseline and 5 years for representative sinus floor elevation magnitudes of 1, 3, and 6 mm. Error bars represent 95% CIs.

The adjusted estimated marginal values for representative elevation magnitudes of 1, 3, and 6 mm were, at baseline, 0.054 mm (95% CI, 0.025–0.084), 0.088 mm (0.071–0.105), and 0.138 mm (0.099–0.178), respectively. At 5 years, the corresponding values were 0.071 mm (0.042–0.101), 0.113 mm (0.096–0.130), and 0.175 mm (0.136–0.215). These adjusted estimates are illustrated in Figure 2.

In the sensitivity analysis using actual follow-up time, the follow-up time × elevation magnitude interaction was B = 0.0007 mm/year per 1-mm increase (SE = 0.0004; 95% CI, −0.0001 to 0.0015; *p* = 0.076), consistent with the primary analysis.

### 3.5. Separation of Baseline Differences from Longitudinal Change

Separate analysis of the baseline time point showed that the gradient associated with elevation magnitude was already present at baseline. In the adjusted GEE model, a 1-mm greater elevation magnitude was associated with a 0.018-mm higher baseline marginal bone level (95% CI, 0.005–0.031; *p* = 0.005).

In the model for marginal bone level at 5 years, no association between elevation magnitude and the final value was detected after mandatory adjustment for baseline marginal bone level and the available covariates (B = 0.000 mm per 1-mm elevation; 95% CI, −0.004–0.005; *p* = 0.842).

Expressing the same model on the longitudinal change scale yielded, as expected, the same estimate for elevation magnitude (B = 0.000 mm per 1 mm; 95% CI, −0.004–0.005; *p* = 0.842). This reparameterization is presented for direct interpretation of marginal bone-level change and does not constitute a statistical analysis independent of the 5-year value model adjusted for baseline level.

Taken together, these analyses indicate that the gradient associated with elevation magnitude was already present at the baseline assessment, with no evidence that elevation magnitude was associated with a subsequent differential longitudinal change in marginal bone level. The results of the primary and secondary models are summarized in Table 5.

Adjusted marginal bone-level values at 5 years were 0.112 mm for a 1-mm elevation, 0.113 mm for 3 mm, and 0.115 mm for 6 mm. Differences among these values were very small after controlling for baseline marginal bone level.

**Figure 3:**
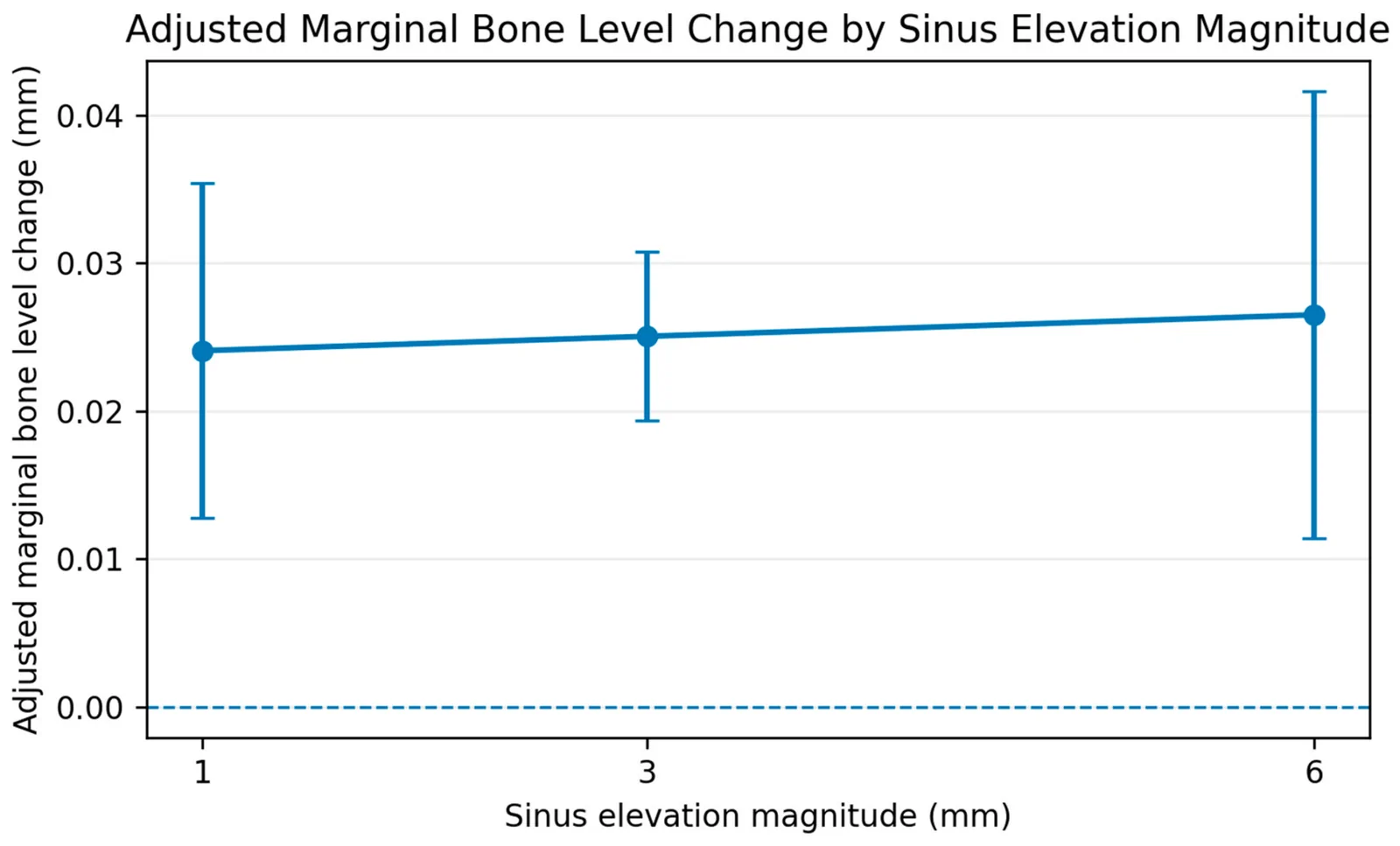
Adjusted change in marginal bone level between baseline and 5 years for representative sinus floor elevation magnitudes of 1, 3, and 6 mm. Error bars represent 95% CIs.

In the longitudinal change analysis, adjusted marginal values were 0.024 mm (95% CI, 0.013–0.035) for 1 mm, 0.025 mm (0.019–0.031) for 3 mm, and 0.027 mm (0.011–0.042) for 6 mm. These adjusted longitudinal changes are illustrated in Figure 3.

### 3.6. Relationship Shape, Effect Modification, and Robustness Analyses

Adding a quadratic term for sinus floor elevation magnitude did not improve modeling of marginal bone level at 5 years or longitudinal change. For the elevation^2^ term, the coefficient was 0.0009 (95% CI, −0.0021–0.0040; *p* = 0.558). For the linear specification, AIC and BIC were −1410.74 and −1355.39, compared with −1409.12 and −1349.51 for the quadratic model; the likelihood-ratio test yielded *p* = 0.540. Consequently, elevation magnitude was retained as a quantitative predictor modeled linearly.

Collinearity diagnostics did not indicate a problem for the primary predictor, with elevation magnitude showing VIF = 1.16 and tolerance = 0.859. The highest VIF values were observed for provisional and definitive retention, at 7.42 and 7.27, respectively. These values indicate moderately increased collinearity between the two prosthetic covariates; therefore, their individual coefficients were not interpreted as independent effects. However, this did not affect collinearity of the primary predictor.

The exploratory effect-modification analysis did not show a significant influence of implant-site preparation technique on the relationship between elevation magnitude and bone outcomes. The technique × elevation magnitude interaction was not significant for either marginal bone level at 5 years (*p* = 0.246) or longitudinal change (*p* = 0.214).

**Figure 4:**
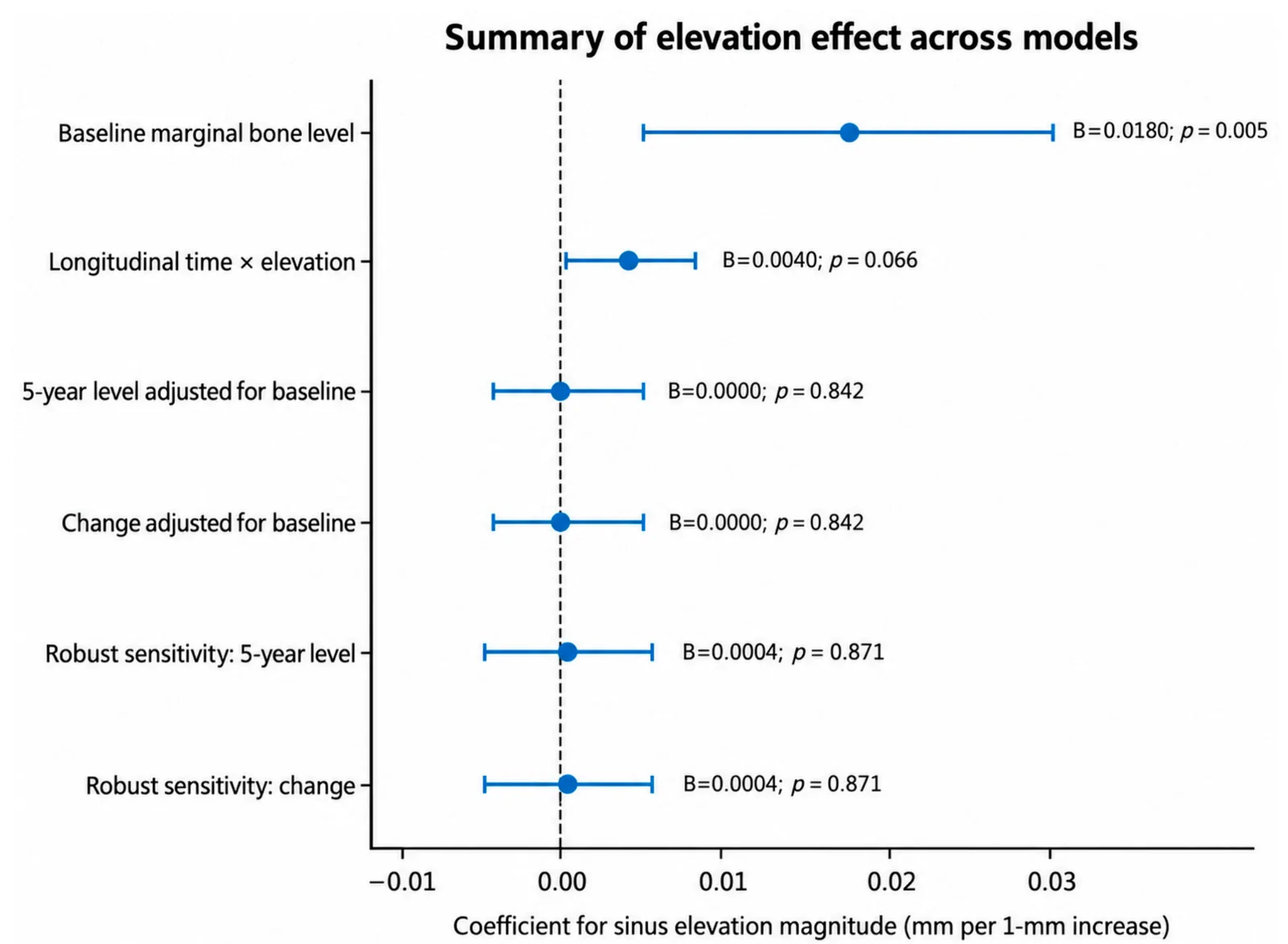
Summary of sinus floor elevation-magnitude coefficients in the primary longitudinal model, secondary models, and sensitivity analyses.

Sensitivity analyses yielded coefficients very close to zero for both marginal bone level at 5 years and longitudinal change. Results were similar whether patient-clustered robust standard errors or patient-clustered bootstrap with 2000 resamples were used. The sensitivity analyses are summarized in Table 6, and the elevation-magnitude coefficients across the primary, secondary, and sensitivity models are shown in Figure 4.

### 3.7. Analytical Sample and Missing Data

Six of the 528 implants did not have a final radiographic assessment because of implant loss. The 522 implants from 247 patients constituted the complete-case sample for all radiographic models. There were no additional missing values for elevation magnitude or for covariates included in the main models, and no data imputation was required.

## 4. Discussion

Within this retrospective cohort and the clinical protocol used, sinus floor elevation magnitude between 1 and 6 mm was not statistically associated with differential marginal bone-level change between the immediate postoperative reference examination and the available assessment performed after at least 5 years. In the primary longitudinal model, a 1-mm greater elevation magnitude was associated with a baseline marginal bone-level value that was 0.017 mm higher, whereas the time × elevation magnitude interaction did not reach statistical significance. In the GEE model for marginal bone level at 5 years, no association with elevation magnitude was detected after adjustment for the baseline value and the available covariates, and expressing the same model on the longitudinal change scale yielded the same estimate.

Importantly, the immediate postoperative examination represents a postsurgical reference rather than a preoperative anatomical baseline. Therefore, the baseline gradient associated with elevation magnitude cannot be attributed specifically to pre-existing anatomy and may reflect a combination of site anatomy, implant positioning, surgical intervention, and immediate postoperative radiographic conditions. Accordingly, the observed associations should be interpreted within the specific clinical protocol used in this cohort rather than as isolated biological effects of elevation magnitude.

This observation is relevant because radiographic parameters obtained after sinus floor elevation describe different biological compartments. Residual bone height, endo-sinus bone gain, and marginal bone level should not be considered equivalent expressions of the same process. Zill et al. showed that baseline bone anatomy may be associated with apical bone formation without showing the same relationship with marginal bone loss [5], whereas long-term randomized data confirmed that substantial changes in the endo-sinus compartment can coexist with limited crestal changes [6]. In addition, the importance of the baseline radiographic value for interpreting subsequent evolution has recently been demonstrated in implant cohorts with long-term follow-up [9].

Studies with long-term follow-up after sinus floor elevation have generally reported favorable implant outcomes across diverse surgical protocols [10,11,12,13,14,15,16,17,18]. However, they predominantly evaluated implant survival and changes in the augmented bone compartment, without directly characterizing the relationship between each additional millimeter of elevation and the longitudinal trajectory of marginal bone level.

Elevation magnitude should be interpreted in the context of baseline anatomy and the procedural protocol. The required elevation is related to residual bone height, sinus morphology, and selection of the surgical approach [19,20,21,22,23,24]. In addition, residual bone height, sinus geometry, Schneiderian membrane characteristics, and the amount of augmentation material were not uniformly available for adjustment in the present cohort. Because these factors are closely related to the clinical indication for sinus elevation and to the magnitude ultimately achieved, substantial residual confounding by baseline anatomy and procedural characteristics remains possible. The estimated coefficients therefore describe associations observed within the measured covariates and the clinical protocol used and should not be interpreted as causal estimates or as the isolated biological effect of increasing sinus elevation by 1 mm.

Studies of augmentation materials and grafting strategies indicate that biomaterial selection, as well as the use or omission of grafting, may influence endo-sinus bone formation and radiographic and clinical outcomes after sinus floor elevation [25,26,27,28,29,30,31,32,33,34,35,36,37,38,39,40]. Schneiderian membrane characteristics and sinus anatomy may also contribute to procedural outcomes [41,42,43,44,45]. These variables were not uniformly available in the present cohort and represent potential sources of residual confounding. At the same time, changes in the endo-sinus compartment cannot be directly extrapolated to the evolution of crestal marginal bone level.

Interpretation of these very small estimated differences must also consider the limitations of the imaging method. The CBCT voxel size used in this study was 0.125 mm, whereas several estimated differences were on the order of only a few hundredths of a millimeter. Although statistical modeling can estimate cohort-level mean differences smaller than the nominal voxel dimension, such estimates should not be interpreted as directly measurable changes of equivalent precision at the individual-implant level. Implant-related artifacts and other limitations of CBCT may further affect peri-implant bone assessment [46,47]. Therefore, the clinical relevance of differences of this magnitude should be interpreted cautiously.

Interpretation of marginal bone level also requires consideration of factors that are not specific to sinus surgery. A recent meta-analysis of studies with a minimum follow-up of 5 years reported differences between implants placed in augmented bone and those placed in native bone, together with substantial between-study heterogeneity [48]. Reviews of risk factors for marginal bone loss indicate that the outcome may be influenced by patient characteristics, periodontal history, smoking, implant characteristics, and peri-implant tissues [49,50,51,52,53,54,55]. A history of periodontitis and smoking have been associated in several meta-analyses with greater marginal bone loss [50,51,52,53,54], whereas soft-tissue phenotype may influence early remodeling [55]. This multifactorial nature supports adjustment of the present models for the available clinical factors and argues against attributing final bone-level differences to a single procedural parameter.

Sensitivity analyses supported the results of the secondary models. Both models with patient-clustered robust standard errors and the clustered bootstrap produced elevation-magnitude coefficients very close to zero for marginal bone level at 5 years and for longitudinal change. Quadratic-term modeling also did not improve model fit, providing no evidence of a nonlinear relationship within the analyzed 1–6 mm range. This consistency across statistical specifications is relevant given the asymmetric distribution of the outcome and the high frequency of values close to zero.

### 4.1. Clinical Relevance of the Study

The main clinical implication is that sinus floor elevation magnitude should not be used in isolation to anticipate long-term marginal bone remodeling. The clinical literature emphasizes that selection of the sinus floor elevation procedure should be based on the overall anatomical conditions, including residual bone height, sinus morphology, the number of planned implants, and the possibility of achieving primary stability [56]. Planning rehabilitation of the atrophic posterior maxilla should integrate residual bone height, sinus morphology, prosthetic requirements, and the available alternatives for augmentation or implant use [42,56,57,58]. Contemporary elevation techniques aim to obtain an adequate bone volume through procedures adapted to the anatomy and clinical context [59,60]. In the present cohort, no longitudinal association between elevation magnitude and marginal bone-level change was detected after adjustment for the available covariates.

Recent data support separating endo-sinus outcomes from peri-implant marginal remodeling. Wang et al. showed that predictors of vertical bone gain and marginal bone loss are not identical [61]. Recent studies of sinus floor elevation with and without biomaterial have reported differences in endo-sinus bone gain without a necessary correspondence with implant or marginal outcomes [62,63]. At the same time, studies with a minimum follow-up of 5 years confirm the multifactorial nature of marginal bone resorption [64], and recent data on transcrestal elevation in sites with 3–5 mm of residual bone report favorable clinical and radiographic outcomes [65]. These findings support the interpretation of the present results, namely that anatomy and the extent of endo-sinus regeneration cannot be used as direct surrogates for longitudinal marginal bone-level change.

The present data allow direct quantification of this implication. After adjustment for baseline bone level, the estimated marginal level at 5 years was 0.112 mm for a 1-mm elevation, 0.113 mm for 3 mm, and 0.115 mm for 6 mm. Adjusted longitudinal change was 0.024, 0.025, and 0.027 mm, respectively. The adjusted differences among these values were very small, with no evidence of a clear longitudinal gradient associated with elevation magnitude within the analyzed range.

A second implication concerns interpretation of radiographic follow-up examinations. A higher marginal bone-level value at a late assessment should not automatically be labeled as greater bone loss if the baseline value is unknown. In the present cohort, the gradient according to elevation magnitude was already present on the immediate postoperative CBCT examination, and after inclusion of this reference value in the model, the association with the 5-year outcome disappeared. This result does not demonstrate that the differences existed preoperatively and does not allow them to be attributed exclusively to baseline anatomy. For clinical monitoring and longitudinal studies, comparison with a reference examination is therefore more informative than isolated interpretation of a single late radiographic value.

The results should not, however, be interpreted as evidence of equivalence across all elevation magnitudes. The conclusion is limited to the 1–6 mm range represented in this cohort and to surviving implants with paired immediate postoperative and long-term radiographic assessments. These data do not show that elevations greater than 6 mm behave similarly, nor that elevation magnitude can replace assessment of residual bone height, sinus anatomy, primary stability, or patient risk factors in treatment planning.

### 4.2. Strengths and Limitations

A strength of the study is the assessment of elevation magnitude on a numerical scale rather than a comparison limited to arbitrary categories. The longitudinal analysis included 522 implants from 247 patients and 1044 observations, and the hierarchical data structure was addressed using random effects for patient and implant. Use of the longitudinal model, the separate baseline-value model, and the 5-year model adjusted for baseline level, together with its re-expression on the change scale, allowed values already present immediately postoperatively to be distinguished from changes observed during follow-up.

The main limitation is the retrospective design. In addition, the available archive did not allow retrieval of the total number of cases initially assessed or the individual reasons for exclusion before formation of the final cohort. Therefore, the complete screening and selection process could not be reconstructed. Although the 60–66-month CBCT examination formed part of the routine clinical follow-up protocol rather than being performed for a specific clinical indication, inclusion in the longitudinal radiographic analysis depended on the availability of this examination. Because the number and characteristics of initially treated patients without an available long-term CBCT, and the reasons for its absence, could not be reconstructed retrospectively, availability-related selection bias cannot be excluded. Elevation magnitude was not assigned experimentally but resulted from the anatomy and surgical requirements of each site. In addition, selection of the surgical approach was linked to the required elevation magnitude, limiting complete separation of the magnitude effect from the procedural context in which it was achieved. Consequently, the study does not allow estimation of a causal effect of elevation magnitude independent of the surgical approach. The amount of augmentation material could not be quantified retrospectively in a uniform manner.

The two prosthetic retention variables showed moderately increased VIF values and were retained only as adjustment variables; their individual coefficients were not used to make inferences regarding independent prosthetic effects.

Residual bone height, sinus geometry, Schneiderian membrane thickness, and the exact amount of augmentation material were not uniformly available for all implants and therefore could not be included as distinct covariates in the adjusted models. These variables are closely related to the anatomical indication for sinus elevation, the required elevation magnitude, and the procedural context in which the elevation was achieved. Consequently, substantial residual confounding by baseline anatomy and procedural characteristics remains possible despite adjustment for the variables available in the dataset. The elevation-magnitude coefficients should therefore be interpreted as conditional associations within this cohort rather than as estimates of an independent or isolated biological effect of elevation magnitude.

The baseline radiographic assessment was also performed immediately after surgery rather than preoperatively. Therefore, the gradient observed at this examination cannot be interpreted as a pre-existing anatomical difference and may reflect the combined effects of baseline anatomy, implant positioning, the surgical procedure, and immediate postoperative radiographic conditions.

Another limitation is the unequal distribution of elevation magnitude. The number of implants was considerably smaller at 5 and 6 mm than at the intermediate values, reducing the precision of estimates at the upper end of the distribution. Consequently, the lack of a significant association should not be interpreted as evidence of biological equivalence across all magnitudes.

The six implants lost before the final assessment could not contribute radiographic values to the 5-year models; therefore, those results represent a complete-case analysis. The single-center design, use of the same implant system, and performance of all procedures by the same clinician reduce operator variability but limit generalizability to other protocols and populations. The timing of the final radiographic assessment was not identical for all implants. Actual follow-up duration was therefore reported descriptively and examined according to elevation magnitude. In addition, a sensitivity analysis repeated the primary longitudinal model using the individual elapsed follow-up time rather than assigning all final examinations to a common 5-year time point. The follow-up time × elevation magnitude interaction remained small and was not statistically significant (B = 0.0007 mm/year per 1-mm increase; 95% CI, −0.0001 to 0.0015; *p* = 0.076), indicating that accounting for the individual follow-up duration did not materially alter the primary longitudinal finding.

Identification of the original sinus-floor reference relied on visual correspondence between the preoperative and immediate postoperative CBCT datasets rather than software-based image registration. Although the planned implant site, local contour of the native sinus floor, and postoperative implant position were used to establish correspondence, some observer-dependent uncertainty in matching the sections and identifying the reference level cannot be excluded. Although baseline and follow-up examinations were acquired using the same CBCT unit and identical acquisition settings, exact reconstruction-related settings and software-version information were not systematically retained in the retrospective archive. Therefore, complete standardization of all post-acquisition image-processing parameters across the follow-up period cannot be confirmed.

Another limitation concerns the radiographic assessment. The CBCT voxel size was 0.125 mm, whereas some differences estimated by the statistical models were smaller than this value. These estimates represent mean differences obtained by modeling a large number of observations and should not be interpreted as changes that can be individualized with the same precision at the level of a single implant. Inter-examiner reproducibility was formally assessed in a random subset of 60 implant sites for both sinus floor elevation magnitude and marginal bone-level measurements. The observed ICCs indicate that measurement variability remains relevant, particularly given the small magnitude of several estimated radiographic differences. The SEM was 0.512 mm for sinus floor elevation magnitude and approximately 0.05 mm for marginal bone-level measurements, emphasizing that differences of only a few hundredths of a millimeter should not be interpreted as precisely measurable changes at the individual-implant level. Importantly, the SEM of 0.512 mm for sinus floor elevation magnitude is substantial relative to the 1-mm increment used to model the exposure. Under the assumptions of classical non-differential measurement error, such imprecision in a continuous exposure may attenuate the estimated association toward the null and reduce the ability to detect an existing association. Accordingly, the non-significant time × elevation magnitude interaction should be interpreted cautiously and does not exclude a true association of small magnitude. Because the final elevation-magnitude values used in the analysis were established by consensus among the examiners, the extent and direction of any attenuation attributable to measurement error cannot be quantified from the available data. Formal intra-examiner reproducibility could not be assessed because repeated measurements by the same examiner in a separate session were not available. Examiners were also aware of the assessment time point during marginal bone-level measurement, although they did not have access to the recorded sinus floor elevation magnitude. Consequently, differences on the order of hundredths of a millimeter should be interpreted primarily in terms of the direction and consistency of the estimates rather than as thresholds of clinical relevance.

## 5. Conclusions

Within this retrospective cohort and the clinical protocol used, sinus floor elevation magnitude between 1 and 6 mm was not statistically associated with differential marginal bone-level change between the immediate postoperative reference examination and the available assessment performed after at least 5 years. Small differences in marginal bone level according to elevation magnitude were already present at the immediate postoperative assessment, and no statistically significant differential longitudinal change was detected.

This conclusion applies to surviving implants with paired radiographic assessments and should not be interpreted as evidence of equivalence among different elevation magnitudes. The findings are limited to the 1–6 mm range represented in this cohort and should not be extrapolated to greater elevation magnitudes.

## Figures and Tables

**Table 1:** Patient-level characteristics according to sinus floor elevation-magnitude categories.

Characteristic	Total	Low 1–2 mm	Moderate 3–4 mm	High 5–6 mm	ASD Low vs. Moderate	ASD Low vs. High	Moderate vs. High
Distinct patients, *n*	248	139	162	53	–	–	–
Age, years	42.00 ± 10.66; 42.00 (33.75–49.25); 21.00–67.00;	41.60 ± 11.41; 41.00 (32.00–50.00); 21.00–65.00;	43.54 ± 9.93; 44.00 (37.00–51.00); 22.00–67.00;	44.53 ± 8.47; 46.00 (38.00–52.00); 27.00–60.00;	0.182	0.274	0.103
Number of implants/patient	2.13 ± 1.01; 2.00 (1.00–3.00); 1.00–4.00; *n* = 248	2.18 ± 1.05; 2.00 (1.00–3.00); 1.00–4.00; *n* = 139	2.38 ± 0.99; 2.00 (2.00–3.00); 1.00–4.00; *n* = 162	2.72 ± 0.86; 3.00 (2.00–3.00); 1.00–4.00; *n* = 53	0.193	0.535	0.354
Female	156 (62.9%)	91 (65.5%)	97 (59.9%)	34 (64.2%)	0.116	0.028	0.088
Male	92 (37.1%)	48 (34.5%)	65 (40.1%)	19 (35.8%)	0.116	0.028	0.088
Smoking: no	171 (69.0%)	103 (74.1%)	101 (62.3%)	33 (62.3%)	0.255	0.256	0.002
Smoking: yes	77 (31.0%)	36 (25.9%)	61 (37.7%)	20 (37.7%)	0.255	0.256	0.002
History of periodontitis: no	201 (81.0%)	102 (73.4%)	135 (83.3%)	49 (92.5%)	0.243	0.524	0.282
History of periodontitis: yes	47 (19.0%)	37 (26.6%)	27 (16.7%)	4 (7.5%)	0.243	0.524	0.282

Note: Patient-level characteristics are based on unique patients. In the Total column, each of the 248 patients is represented once. Within each elevation-magnitude category, a patient is represented once if at least one implant belonged to that category. Because the same patient could contribute implants to more than one elevation category, the patient counts across categories are not mutually exclusive and should not be summed. ASD, absolute standardized difference.

**Table 2:** Implant-level clinical, procedural, and radiographic characteristics according to sinus floor elevation-magnitude categories.

Characteristic	Total	Low 1–2 mm	Moderate 3–4 mm	High 5–6 mm	ASD Low vs. Moderate	ASD Low vs. High	ASD Moderate vs. High
Implants, *n*	528	205	261	62	–	–	–
Follow-up duration, months	63.94 ± 1.06; 64.00 (63.00–65.00); 63.00–66.00;	63.32 ± 0.66; 63.00 (63.00–63.00); 63.00–66.00;	64.24 ± 1.12; 64.00 (63.00–65.00); 63.00–66.00;	64.78 ± 0.67; 65.00 (64.00–65.00); 64.00–66.00;	–	–	–
Baseline marginal bone level, mm	0.09 ± 0.15; 0.00 (0.00–0.20); 0.00–0.62;	0.07 ± 0.14; 0.00 (0.00–0.00); 0.00–0.62;	0.09 ± 0.15; 0.00 (0.00–0.20); 0.00–0.52;	0.14 ± 0.20; 0.00 (0.00–0.31); 0.00–0.61;	–	–	–
Marginal bone level at 5 years, mm	0.11 ± 0.18; 0.00 (0.00–0.22); 0.00–0.80;	0.09 ± 0.16; 0.00 (0.00–0.20); 0.00–0.72;	0.12 ± 0.17; 0.00 (0.00–0.23); 0.00–0.62;	0.17 ± 0.23; 0.00 (0.00–0.36); 0.00–0.80;	–	–	–
Marginal bone-level change, mm	0.03 ± 0.06; 0.00 (0.00–0.00); −0.01–0.51;	0.02 ± 0.06; 0.00 (0.00–0.00); −0.01–0.51;	0.03 ± 0.06; 0.00 (0.00–0.00); −0.01–0.32;	0.03 ± 0.06; 0.00 (0.00–0.00); 0.00–0.31;	–	–	–
Technique: Osteotome	204 (38.6%)	79 (38.5%)	101 (38.7%)	24 (38.7%)	0.003	0.004	0.000
Technique: Versah	171 (32.4%)	67 (32.7%)	82 (31.4%)	22 (35.5%)	0.027	0.059	0.086
Technique: DASK Simple	153 (29.0%)	59 (28.8%)	78 (29.9%)	16 (25.8%)	0.024	0.067	0.091
Definitive retention: cemented	162 (30.7%)	68 (33.2%)	79 (30.3%)	15 (24.2%)	0.062	0.199	0.137
Definitive retention: screw-retained	366 (69.3%)	137 (66.8%)	182 (69.7%)	47 (75.8%)	0.062	0.199	0.137
Provisional retention: cemented	175 (33.1%)	75 (36.6%)	84 (32.2%)	16 (25.8%)	0.093	0.234	0.141
Provisional retention: screw-retained	353 (66.9%)	130 (63.4%)	177 (67.8%)	46 (74.2%)	0.093	0.234	0.141
Immediate restoration	450 (85.2%)	149 (72.7%)	241 (92.3%)	60 (96.8%)	0.536	0.711	0.197
Immediate loading	78 (14.8%)	56 (27.3%)	20 (7.7%)	2 (3.2%)	0.536	0.711	0.197
Implant lost: no	522 (98.9%)	203 (99.0%)	259 (99.2%)	60 (96.8%)	–	–	–
Implant lost: yes	6 (1.1%)	2 (1.0%)	2 (0.8%)	2 (3.2%)	–	–	–

Note: Implant-level characteristics are based on 528 implants. Follow-up duration and radiographic outcomes are reported for the 522 implants with paired baseline and long-term CBCT assessments; the corresponding numbers were 203 implants in the low-elevation category, 259 in the moderate-elevation category, and 60 in the high-elevation category. Implant loss is reported for the full eligible cohort of 528 implants. ASD values were calculated for baseline patient, clinical, and procedural characteristics and were not calculated for radiographic outcomes or implant loss. ASD, absolute standardized difference.

**Table 3:** Baseline and 5-year marginal bone levels and longitudinal change according to exact sinus floor elevation magnitude.

Sinus Floor Elevation Magnitude, mm	Evaluated Implants, n	Patients, n	Baseline Bone Level, Mean ± SD	Baseline Bone Level, Median (IQR); Min–Max	Bone Level at 5 Years, Mean ± SD	Bone Level at 5 Years, Median (IQR); Min–Max	Bone-Level Change, Mean ± SD	Bone-Level Change, Median (IQR); Min–Max
1	54	48	0.05 ± 0.13	0.00 (0.00–0.00); 0.00–0.51	0.07 ± 0.14	0.00 (0.00–0.10); 0.00–0.51	0.02 ± 0.05	0.00 (0.00–0.00); 0.00–0.27
2	149	106	0.08 ± 0.14	0.00 (0.00–0.13); 0.00–0.62	0.10 ± 0.17	0.00 (0.00–0.20); 0.00–0.72	0.02 ± 0.07	0.00 (0.00–0.00); −0.01–0.51
3	125	97	0.05 ± 0.12	0.00 (0.00–0.00); 0.00–0.50	0.07 ± 0.14	0.00 (0.00–0.00); 0.00–0.60	0.01 ± 0.05	0.00 (0.00–0.00); 0.00–0.31
4	134	80	0.12 ± 0.17	0.00 (0.00–0.26); 0.00–0.52	0.16 ± 0.19	0.00 (0.00–0.31); 0.00–0.62	0.04 ± 0.07	0.00 (0.00–0.02); −0.01–0.32
5	44	40	0.12 ± 0.18	0.00 (0.00–0.29); 0.00–0.53	0.14 ± 0.22	0.00 (0.00–0.31); 0.00–0.72	0.02 ± 0.05	0.00 (0.00–0.00); 0.00–0.19
6	16	16	0.19 ± 0.22	0.07 (0.00–0.32); 0.00–0.61	0.24 ± 0.25	0.26 (0.00–0.40); 0.00–0.80	0.05 ± 0.09	0.00 (0.00–0.06); 0.00–0.31
Total	522	247	0.09 ± 0.15	0.00 (0.00–0.20); 0.00–0.62	0.11 ± 0.18	0.00 (0.00–0.22); 0.00–0.80	0.03 ± 0.06	0.00 (0.00–0.00); −0.01–0.51

**Table 4:** Longitudinal linear mixed model for peri-implant marginal bone level.

Predictor	B Coefficient	Standard Error	95% CI	*p*
Time: 5 years versus baseline	0.013	0.007	−0.001–0.027	0.076
Sinus floor elevation magnitude, per 1 mm	0.017	0.006	0.005–0.029	0.006
Time × sinus floor elevation magnitude	0.004	0.002	0.000–0.008	0.066

**Table 5:** Summary of the association between sinus floor elevation magnitude and bone outcomes in the primary longitudinal model and secondary models.

Model	Reported Effect	B	95% CI	*p*	Interpretation
Longitudinal model	Time × elevation interaction	0.004	0.000–0.008	0.066	Weak positive trend, not statistically significant
5-year model adjusted for baseline level	Elevation, per 1 mm	0.000	−0.004–0.005	0.842	No association detected after baseline and covariate adjustment
Baseline-level model	Elevation, per 1 mm	0.018	0.005–0.031	0.005	Statistically significant baseline gradient
Reparameterization on the longitudinal change scale	Elevation, per 1 mm	0.000	−0.004–0.005	0.842	Same estimate as the 5-year model adjusted for baseline level; does not represent an independent analysis

**Table 6:** Robustness analysis for the asymmetric distribution of marginal bone level.

Model	Dependent Outcome	Method Used	B for Elevation	SE/Bootstrap Error	95% CI	*p*	Conclusion
Sensitivity model	Marginal bone level at 5 years	Linear regression with patient-clustered robust standard errors	0.0004	0.0026	−0.0046–0.0055	0.871	No significant association
Sensitivity model	Marginal bone level at 5 years	Patient-clustered bootstrap, 2000 resamples	0.0004	0.0026	−0.0048–0.0056	0.874	No significant association
Sensitivity model	Marginal bone-level change	Linear regression with patient-clustered robust standard errors	0.0004	0.0026	−0.0046–0.0055	0.871	No robust association with longitudinal change
Sensitivity model	Marginal bone-level change	Patient-clustered bootstrap, 2000 resamples	0.0004	0.0025	−0.0049–0.0053	0.853	No robust association with longitudinal change

## Data Availability

The de-identified data supporting the findings are available from the corresponding author on reasonable request, subject to ethical and institutional requirements.
